# Positive exercise test?

**DOI:** 10.1007/s12471-016-0864-0

**Published:** 2016-07-21

**Authors:** W. K. den Dekker, J. W. Deckers, S. C. Yap

**Affiliations:** Department of cardiology, Thoraxcenter, ErasmusMedical Center, Rotterdam, The Netherlands

## Answer

**Fig. 1:**
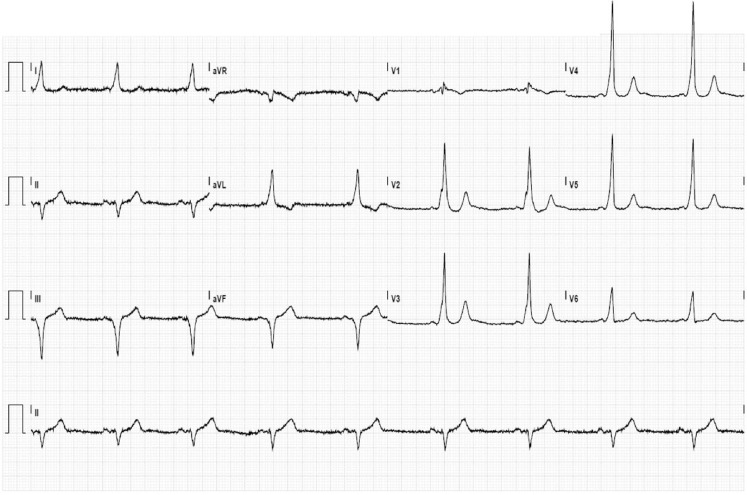
Baseline ECG

The ECG during peak exercise shows sinus tachycardia of 167 beats/min with pre-excitation and ischaemic-like ST-segment depression in V3–V6 (Fig. 1a in the Question). The ECG in the recovery phase shows disappearance of pre-excitation with normalisation of ST segments at a heart rate of 145 beats/min (Fig. 1b in the Question). Pre-excitation reoccurred at a heart rate of 101 beats/min. The baseline ECG more clearly shows pre-excitation consistent with a posteroseptal accessory pathway (Fig. 1). The observation that pre-excitation was still present at peak exercise can be explained by improvement in the anterograde conduction of the accessory pathway during higher adrenergic activity during exercise. The exercise test was regarded inconclusive for the presence of ischaemia.

False-positive ST-segment depression during exercise testing occurs in half of patients with pre-excitation [1, 2]. Nuclear stress perfusion imaging has been proposed as an alternative for ischaemia detection, but this has also been associated with false-positive results [2]. The mechanism of perfusion defects in patients with pre-excitation could be the result of ventricular asynchrony leading to differences in perfusion. Our patient was considered at low risk for significant coronary artery disease, considering his age, absence of risk factors and excellent exercise capacity without ischaemic symptoms.

**Fig. 2:**
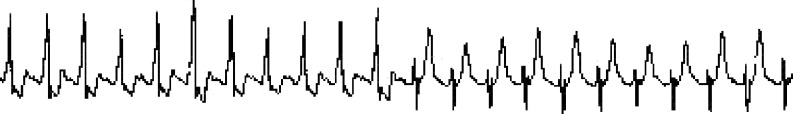
Sudden loss of pre-excitation

The guidelines consider abrupt loss of pre-excitation during exercise testing useful to identify patients at low risk of rapid conduction over the accessory pathway, and thus at low risk for sudden arrhythmic death [3]. Our patient showed abrupt loss of pre-excitation during the recovery phase (Fig. 2). In consideration of the patient’s symptomatic presentation, he was offered an electrophysiology study, which will be performed soon.
